# “*It was a new concept to talk about periods at the state capitol”*: a mixed methods implementation-as-usual evaluation of Georgia's menstrual health and hygiene policy

**DOI:** 10.3389/frph.2025.1745263

**Published:** 2026-01-12

**Authors:** April M. Ballard, Emily Wallace, Pranitha Kaza, Claire Cox, Adele Stewart, Shannon R. Self-Brown

**Affiliations:** 1 Department of Population Health Sciences, Georgia State University School of Public Health, Atlanta, GA, United States; 2 Department of Health Policy and Behavioral Sciences, Georgia State University School of Public Health, Atlanta, GA, United States; 3 Department of Chemistry and Biochemistry, Georgia Institute of Technology, Atlanta, GA, United States; 4 Georgia Stop Tax on Menstrual Products, Macon, GA, United States

**Keywords:** adolescent health, implementation, menstrual equity, menstrual policy, period poverty

## Abstract

**Introduction:**

Since 2017, 32 US states have enacted policies to increase menstrual material access in schools. Yet, the implementation and equity of these efforts remain poorly understood. This mixed methods study evaluated the implementation-as-usual (IAU) processes of Georgia's statewide MHH policy, the first in the US to establish recurring appropriations for menstrual materials in public schools without mandating their provision.

**Methods:**

We conducted document reviews and key informant interviews (KIIs) with state, district, and school-level stakeholders to evaluate IAU. Using framework analysis, we identified and described the core implementation components guided by Quality Implementation Framework and Interactive Systems Framework for Dissemination and Implementation.

**Results:**

Georgia's funding-based approach facilitated administrator buy-in, signaled state support, and enabled local adaptation across contexts. Advocacy groups filled key capacity gaps by providing technical assistance, training, and feedback to policymakers, which helped sustain and expand appropriations. However, limited programmatic guidance led to variability in implementation, communication gaps, and menstrual material access across schools.

**Discussion:**

Findings illustrate the trade-offs between flexibility and accountability in statewide MHH policy design. Appropriations without mandates can enhance local ownership but require complementary structures for guidance and monitoring to ensure equitable and effective implementation.

## 1 Introduction

Managing menstruation in school settings is essential to ensuring that adolescents can learn, thrive, and participate with dignity. Yet globally, students face persistent challenges to safe and hygienic menstrual health and hygiene (MHH) management in school settings (1–6). Limited access to menstrual materials, inadequate water and sanitation infrastructure, and insufficient MHH education are common barriers (1, 7–10). These challenges can negatively affect students' health, psychosocial wellbeing, and school engagement, contributing to absenteeism, stigma, and inequitable educational outcomes (1, 3, 11). Addressing MHH in schools is therefore critical not only for health and hygiene but also for gender equity, educational attainment, and human rights (1, 3, 7, 12–14).

In the United States (US), research on MHH in schools remains limited (15, 16), but a growing body of evidence documents the challenges adolescents face during the school day (1, 7, 14, 17–20). Students report missing school due to lack of access to menstrual materials and experiencing disruptions related to discomfort, odor concerns, and fear of leaks. Many also describe embarrassment or shame when forced to request materials from staff, especially when materials are only available from a nurse or administrator (7, 14, 17, 18, 21). These experiences are often compounded for Black, Indigenous, and other students of color, who may encounter lower levels of menstrual preparedness and higher levels of stigma (17, 22). Without targeted support, school environments that are unsupportive of MHH can therefore reinforce broader racial and economic inequities—impacting students' education, health, and psychosocial development during a formative life stage.

Legislation has emerged as a key tool for improving MHH in school settings (4, 5, 23–26). Some countries have adopted national policies addressing multiple aspects of MHH, while others lack formal policies or have developed narrower strategies, such as removing taxes on menstrual materials or requiring their availability in public facilities (4, 5). In the US, progress has largely occurred at the state level (25–28): between 2017 and 2025, 32 states enacted policies to expand access to free menstrual materials in K-12 schools (26). These legislative gains reflect growing political and public recognition of MHH as an equity issue. However, little is known about how these policies are implemented in practice—a critical gap given the rapid pace of legislative adoption. Without adequate planning, monitoring, and evaluation, policies may not be evidence-based or may fail to achieve equitable, sustainable impacts. To date, only one formal study in the Chicago Public Schools has examined the real-world implementation of MHH policies in US schools (29). A small number of additional descriptive or practice-based assessments have documented aspects of implementation in select areas (e.g., Minnesota; Jackson County, Missouri) (30, 31).

This mixed methods study addresses this gap by examining the implementation-as-usual (IAU) processes of Georgia's (GA's) statewide MHH policy. Evidence from such an evaluation can identify opportunities for improvement and inform the development of tools to strengthen adoption, implementation, and sustainment (32, 33). Notably, GA was the first state in the US to establish recurring appropriations for menstrual materials in public schools without mandating their provision in 2019. Unlike mandates, GA's policy allocates funds without requiring schools to provide menstrual materials, creating a unique opportunity to study implementation variability across districts and schools. Findings from this study can inform implementation support strategies in GA and provide lessons for the 19 states currently funding menstrual materials in schools through appropriations or funded mandates, and the 30 yet to do so (26).

## 2 Methods

### 2.1 Study design and conceptual basis

We conducted key informant interviews (KIIs) and document reviews to evaluate the GA IAU processes of state appropriations legislation to fund free menstrual materials in public schools. The IAU evaluation examined naturally occurring implementation efforts, with an aim to identify effective strategies, barriers, and facilitators; opportunities for improvement; and inform development of tools to support adoption, implementation, and sustainment (32, 33). This study is reported in accordance with the Good Reporting of a Mixed Methods Study (GRAMMS) criteria (34) and the Consolidated Criteria for Reporting Qualitative Research (COREQ) (35). The location(s) where each of the six GRAMMS and 32 COREQ items is provided as Supplementary Material.

We used two conceptual and implementation frameworks as the basis of this study: the Interactive Systems Framework for Dissemination and Implementation (ISF) (36) and the Quality Implementation Framework (QIF) (37). The ISF details structures and functions that work bi-directionally and at multiple levels (e.g., organizational, community) to bridge science and practice (36). This conceptual framework is particularly suitable in contexts with varied and unique structures. It has been successfully used to evaluate the implementation of policies (38–41) and school programs (40, 42–45). The ISF directed our examination of the implementation system and context at the state, district, and school levels. It also helped ensure that all aspects of the implementation system and context were considered, including the Synthesis and Translation System (adapts or translates evidence and innovations), Support System (builds capacity and provides various implementation supports), and Delivery System (carries out implementation).

The QIF synthesizes 25 IS frameworks to identify specific actions that improve the quality of implementation. It is organized into four temporal phases that serve as a roadmap for implementation in community settings: (1) identifying initial considerations about the setting, (2) creating an implementation structure, (3) creating an ongoing implementation structure, and (4) improving future applications (37). Using the QIF phases, we examined the implementation processes since the policy's inception, identified strengths and weaknesses of current implementation, and determined specific actions that may improve the quality of implementation.

### 2.2 Setting

This study was conducted in GA, a southeastern US state with significant economic and health disparities that shape the school environment. Approximately 17% of children under age 18 in GA live in poverty, placing the state in the 65th percentile for child poverty nationally (46). Ability to safely and hygienically manage MHH is constrained by taxation: under current state law, menstrual materials are subject to the state's 4% sales tax, in addition to local taxes (47), creating an added financial burden for low-income individuals who menstruate.

During the 2024–2025 school year, GA's K-12 public school system enrolled approximately 652,000 students who menstruate (48). GA schools operate within a resource-constrained context. In 2024, the full-time school nurse-to-student ratio was 1:1,041, and the school nursing support staff-to-student ratio was 1:2,914 (49), both of which are substantially higher than the National Association of School Nurses' recommended ratio of 1:750 (50). Other student support staff-to-student ratios were also suboptimal, including family services/parent coordinators (1:1,497), school psychologists (1:2,150), and school social workers (1:1,911) (49).

### 2.3 Key informant interviews

#### 2.3.1 Sample and participant selection

To understand how the policy is implemented, we conducted KIIs. To ensure that the multiple actors and processes involved were captured and that diverse points of view were considered, we enrolled representatives from key implementation and advocacy organizations, and district and school nurses, social workers, and counselors who are involved in a range of relevant implementation activities. District and school staff were selected purposively to capture GA's varied geographic regions and economic conditions. Initial key informants were identified with assistance from community partners and recruited at in-person events and via email. Additional participants were recruited through snowball sampling, where interviewees referred other relevant community members, as needed.

Our original study design called for 16–24 interviews (51, 52). The final sample consisted of 13 key informants, which included two MHH state advocates, a state-level implementer, two district-level directors of health services, five lead or school nurses, one school social worker, and one homeless and foster care program liaison (Table 1). The final number was lower than anticipated because interviews began to yield repetitive information, suggesting thematic saturation had been reached (52).

**Table 1:** Key informant interview and document review sample description.

Description	n
**Key informant interview sample**	**13**
Menstrual health and hygiene state advocate	2
State-level implementer	1
District-level director of health services	2
Lead or school nurse	5
School social worker	1
Homeless and foster care program liaison	1
**Document review sample**	**33**
Legislative appropriation documents	6
Georgia Department of Education allocation reports	5
Georgia Department of Education expenditure reports	4
GA Stop Tax on Menstrual Products' website posts	16
GA Stop Tax on Menstrual Products' informal questionnaires with school nurses	2

Bold text represents overall sample type and size for the two data source.

Recruitment efforts extended to 21 additional individuals. Of these, five expressed interest but interviews could not be scheduled due to competing demands. Two individuals affiliated with a charter school responded to indicate that their students received primarily virtual instruction and were therefore not an appropriate fit for the study. Six individuals declined participation, and eight did not respond. Recruitment and scheduling challenges were compounded by the demanding workloads of school-based staff, particularly nurses, social workers, and counselors, who often face understaffing and limited flexibility to step away from student-facing responsibilities. Additionally, district-level email security systems may restrict or block messages from external senders, further limiting outreach success.

#### 2.3.2 Data collection

Author AMB, who has expertise in qualitative research and MHH, conducted all KIIs from February to November 2024. To understand implementation context, components, and actors, we asked key informants about their knowledge of and experiences with the history of GA's MHH policy, implementation processes and strategies, stakeholder roles and responsibilities, and monitoring and evaluation. Probes queried details about barriers and facilitators to implementation and potential solutions to improve implementation. While guides covered the consistent domains across all participant groups, they were tailored slightly to reflect respondents' professional roles and organizational perspectives. All semi-structured interview guides are available in the Supplementary Materials.

Interviews were conducted virtually or by phone. Online interviews were transcribed using artificial intelligence (AI). AI-generated transcripts were manually checked for quality, corrected, and de-identified by our research team. Detailed notes were taken on the contents of phone-based interviews. Interviews lasted between 20 and 60 min. Key informants did not receive compensation for their participation.

### 2.4 Document reviews

#### 2.4.1 Sample

After KIIs were complete, to understand past and present implementation procedures and actors, we also conducted document reviews focusing on materials that described the policy, implementation procedures, resource allocation, and the roles of key actors. The sample included legislative appropriations documents (*n* = 6); DoE allocation (*n* = 5) and expenditure (*n* = 4) reports; GA Stop Tax on Menstrual Products' (STOMP) website posts (*n* = 16); and GA STOMP informal questionnaires conducted with school nurses at Georgia Association for School Nurses (GASN) annual meetings (*n* = 2) (Table 1). We identified legislative documents and GA STOMP website posts via online searches, while DoE allocation and expenditure reports and GA STOMP questionnaires were obtained through direct requests.

#### 2.4.2 Data extraction

We systematically extracted data from all reviewed documents (aside from allocation reports) using a structured table organized around the QIF and ISF (Supplementary Material). For narrative documents, extraction focused on the ISF-relevant actors and systems (e.g., delivery system, support system), QIF implementation phase and related activities (e.g., readiness assessment, capacity building), and potential alignment or divergence with KII data. Document reviews were used to complement and triangulate KII data (53).

Allocation reports from fiscal years 2019–2024 were extracted and analyzed separately due to their quantitative nature. Each Excel-based report included the funding amount allocated to each school and district, each school's grade range, and the estimated number of enrolled students who menstruate. Fiscal years 2019–2021 also included the number of directly certified students, which include students living in a family unit receiving Supplemental Nutrition Assistance Program food stamp benefits, Temporary Assistance for Needy Families benefits, or a Medicaid income that does not exceed free or reduced-price lunch eligibility standards—or students identified as experiencing houselessness, unaccompanied youth, foster children, or migrants (54). Data from these reports were used to reconstruct and verify the allocation formula and to explore how funds were distributed across school types and time.

### 2.5 Data analysis

Qualitative data from KIIs and document reviews, and quantitative data from allocation reports, were analyzed separately and then integrated to comprehensively describe IAU processes. To understand how the allocation process functioned over time, we analyzed allocation report data and reverse-engineered the underlying formulas by comparing funding levels with school grade range, student enrollment, and the number of directly certified students.

To explore other policy processes, we used a framework approach to analyze KII verbatim transcripts, notes, and process documents with MAXQDA 2022 software (VERBI Software, Berlin, Germany). Framework analysis involves five steps: data immersion, development of a thematic framework, coding, charting, and interpretation (55, 56). This approach enabled us to identify connections between data and our pre-determined frameworks, the ISF and QIF.

We developed and iteratively refined a codebook with deductive and inductive codes, informed by the ISF, QIF, existing literature, transcript reading, and debriefing notes. To ensure consistency and reliability in our coding process, we double-coded two transcripts and collaboratively reviewed the coding strategies and interpretation of data by each coder (57). Transcripts were then double coded in sets of three, with author AMB reviewing coding decisions and resolving discrepancies after each round. Team meetings were held throughout the process to systematically debrief, discuss coding differences, and document emerging themes in analytical memos. We did not calculate inter-rater agreement statistics, as the goal of coding was thematic exploration rather than exact agreement and because differences in coding style can result in a low artificial agreement (58, 59).

We assessed both code and meaning saturation throughout the coding process by tracking the number of new codes generated and modifications to existing code definition after each round of coding (i.e., every 2–3 transcripts) (51, 52). Code saturation was considered achieved when 90% of meaningful codes had been identified and developed, which occurred after four transcripts. Meaning saturation was considered met when 90% of core codes had fully developed characteristics, which occurred after seven transcripts. Following coding, we conducted a structured, multi-phase analysis using summation tables and analytical memos to synthesize and interpret findings (51, 55, 56). We began by generating detailed memos for each QIF phase and sub-phase by querying relevant codes and intersections of codes. These summaries captured both recurring themes and notable absences where QIF steps may not have been completed. Next, we identified and described the core implementation components at the state, district, and school levels drawing on the QIF and ISF as guiding constructs. Lastly, we examined the broader implementation system using the ISF to map actor roles and interactions across system levels, highlighting both formal structures and informal dynamics influencing implementation.

### 2.6 Ethics

All participants provided informed, verbal consent prior to data collection and received a copy of the consent form. A waiver of signature was requested and approved because the study was minimal risk and it was not possible to obtain written consent from all participants, since interviews occurred virtually or over the phone. Participants were made aware of their right to skip any questions and end interviews at any time. The Georgia State University Institutional Review Board approved all study procedures.

## 3 Results

**Figure 1:**
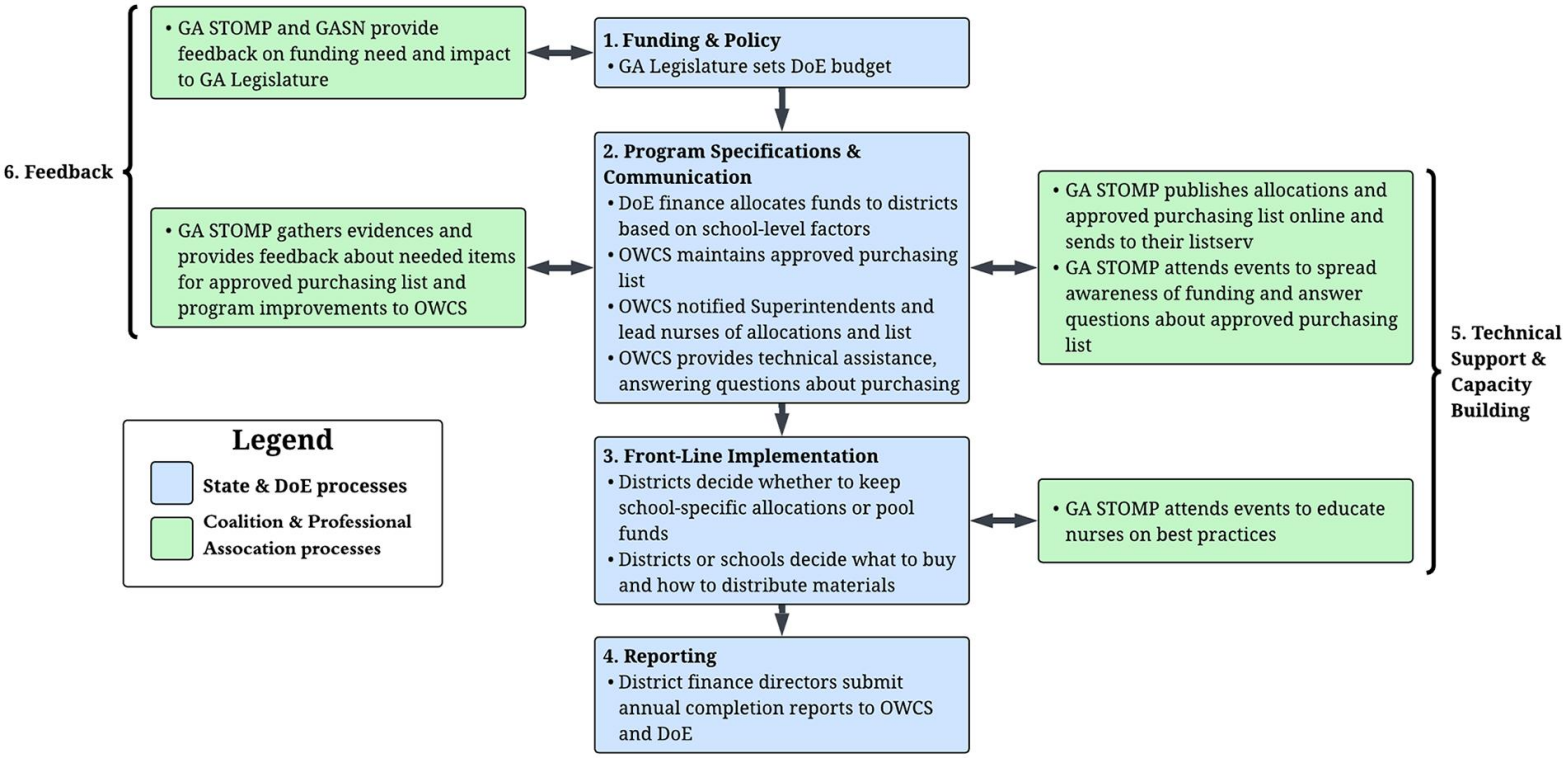
Overview of the core components of the GA's MHH appropriations legislations. DoE, Department of Education; OWCS, Office of Whole Child Support; GA STOMP, GA Stop Tax on Menstrual Products; GASN, GA Association of School Nurses.

Figure 1 provides a high-level overview of GA's MHH appropriations legislation, illustrating the six core implementation components as identified through document reviews and KIIs: (1) funding and policy administration through the GA Legislature, (2) program specifications and communication by DoE and the Office of Whole Child Support (OWCS), (3) front-line implementation via districts and schools, (4) finance directors reporting to OWCS and DoE, (5) GA STOMP facilitating technical support and capacity building, and (6) GA STOMP and GASN providing feedback to the GA Legislature and OWCS. These processes evolved over time and are described throughout the following sections using the four temporal QIF phases: emergence of GA's MHH policy and initial considerations regarding GA, creating a structure for implementation, ongoing implementation support strategies, and improving future applications. Across these sections, we also highlight how key actors and organizations performed functions aligned with the ISF—including Synthesis and Translation, Support, and Delivery System roles—to bridge guidance, evidence, and practice across the state, district, and school levels.

Since the policy's passing in 2019, GA STOMP (a state coalition) worked closely with the GA Legislature, DoE, GASN (a school nurse professional association), and district and school nurses, transforming into implementation champions—volunteers or appointees that actively promote and facilitate implementation. As champions, the state coalition developed politically savvy strategies to sustain and increase funding, led education and feedback efforts with front-line implementers, promoted best practices for menstrual material distribution in schools, and publicized the DoE-established policy requirements. These championing efforts ultimately created conditions for adaptation, capacity-building, and sustainment.

### 3.1 Emergence of GA's MHH policy and initial considerations regarding GA

#### 3.1.1 Emergence of GA's MHH policy

Due in large part to GA STOMP's advocacy, GA became the first state to pass a policy funding menstrual materials in schools without mandating their provision in 2019. However, school-based MHH was not the group's initial focus, nor was an appropriations policy their goal. GA STOMP, formed in 2017, initially focused on removing the 4% sales tax applied to menstrual materials, recognizing the tax as an added financial burden for low-income individuals who menstruate. Through this early advocacy, the coalition identified other critical issues related to MHH, including gaps in menstrual material provision in schools, prisons, and during emergencies or natural disasters.

As their involvement and focus expanded to include school-based MHH, the advocacy group shared anecdotal reports from school nurses, students, and local organizations with legislators to highlight the issue of period poverty, its impact on Georgia students, and the need for menstrual materials in schools. Before GA enacted the appropriations policy, nurses and other school staff across the state were routinely using personal funds to purchase menstrual materials for students.

…68% of the time, period products were being paid for either out of a school nurse's pocket or administrators or principals at school. So, they knew that these products were needed most. School budgets didn't have the money to buy them, so they were just doing what women do, helping each other out and going and buying it. (MHH advocate 1)

According to key informants, GA STOMP's advocacy—combined with legislators' personal connections with those who had experienced period poverty—led to the passing of GA's MHH legislation. Politically, some viewed the appropriations legislation as a way for the state to acknowledge period poverty as a significant issue without removing the sales tax on menstrual materials. While this outcome was not the advocates' original goal, it was seen as a major accomplishment and a sign of progress, especially in light of initial resistance to even discussing menstruation in policymaking spaces.

I think early on there was a lot of resistance and just not wanting to discuss [periods] … It was kind of a new concept to talk about periods at the State Capitol  … But almost without exception, individual conversations were had, and needs were laid out and understanding was had. We have found a fairly receptive audience with state leaders and legislators. (MHH advocate 1)

#### 3.1.2 Initial considerations regarding GA

Because neither advocates nor legislators had originally planned to pursue an appropriations policy, no formal needs, resource, fit, or capacity assessments were conducted. Instead, the policy was initiated quickly, and funding was allocated with a “try it and see” approach. This left little time or guidance for establishing an implementation structure. However, according to MHH advocates and the state-level implementer, this approach was seen as a great fit for GA and was highly favorable to state mandates, which were seen as conflicting with GA's prevailing “local control” ethos and would likely to be met with resistance if funding was not attached.

I think the difference between [a state that prioritizes] local control vs. those that do not is that if you're local control and you're going to mandate something, you better put some dollars with it … If you don't, it's going be hard to pull it off. (State-level implementer)

On paper, the policy was intended to increase access to menstrual materials in schools for low-income or economically disadvantaged students. The legislation created an automatically renewing funding stream that increased over time ($960K in 2019 to $1.45 million in 2024). Document reviews showed that the original state budget allocated “funds for grants to schools for feminine hygiene products for low-income students” (HB 31 FY2020G 149.8, page 102). Budgets in fiscal years 2021 (HB 793) and 2022 (HB 81) specified that school systems should be prioritized based on low property tax wealth and a high percentage of economically disadvantaged students. However, additional details were not legislatively documented or publicly available because of the nature of allocations processes.

After the policy was passed, GA DoE prioritized capacity building in collaboration with GA STOMP. Together, they launched outreach and educational efforts targeting district- and school-level actors, particularly administrators and nurses to foster buy-in. In 2020, they co-hosted a statewide webinar that targeted school nurses, who were intentionally selected by GA DoE and GA STOMP as the primary implementers of the policy in schools. The webinar educated nurses on the policy, outlined how to access funds, and aimed to both build general capacity and explicitly recruit nurses into an implementation role.

GA STOMP has actively been a part of training school nurses. Early on, it was just about the existence of the money and to ask their [district or school] finance person or their school superintendent about it … When the money first went out, we did a lot of emailing, creating flyers. We created a best practices thing that went along with [DoE's] email to school superintendents. Then, the following February of 2020, right before COVID, we had a school nurse webinar at DoE … and that was recorded for other school nurses to be able to watch. (MHH advocate 1)

GA STOMP continued to serve as an implementation champion by participating in state-level nursing conferences and continuing education events to publicize the policy and promote best practices for menstrual material distribution in schools. These activities helped foster buy-in from school-level implementers and created a more supportive climate for policy uptake.

### 3.2 Creating a structure for implementation

Upon passage, there were a range of activities required of the DoE to operationalize the policy. Because neither advocates nor legislators had initially anticipated an appropriations policy, there was little time to create a formal implementation team before the policy took effect. State, district, and school-based teams and processes were therefore initially established hastily and later refined to better fit the context, which is described further in **Section 3.3. Ongoing implementation support strategies**. The implementation team and processes evolved over time and were spread across sectors (e.g., government, schools, advocacy organizations) to address resource gaps and practice-based needs. Table 2 summarizes the key organizations and actors involved in implementation and outlines their roles across state, district, and school levels, and how those roles fit into the ISF.

**Table 2:** Key implementation actors and roles.

Level	Organization/Actor	Role in implementation
ISF Synthesis and Translation System		
State	Georgia (GA) Department of Education (DoE)—Finance staff and contractor^a^	Developed allocation formula Managed funds Served as initial contact for nurses
	GA STOMP^a^	Co-led outreach Trained nurses Promoted buy-in Developed materials and best practices
ISF Support System		
State	GA DoE—Full-time program director with school nurse expertise	Served as central coordinator of policy Strengthened communication Provided technical assistance
	GASN	Provided support and dissemination among nursing community
ISF Delivery System		
District	District administrators (e.g., superintendents, finance personnel)	Informed staff of funds Oversaw fund access Supported school-level implementation
	District or lead nurses	Acted as intermediaries between state guidance and school-level action, but sometimes also served as in-school nurses
School	School administrators	Facilitated program logistics within schools
	School nurses (targeted front-line implementers)	Distributed menstrual materials Monitored needs Engaged in training and adaptation

a Played a role in both the Synthesis and Translation System and the Support System.

#### 3.2.1 State-level team and plan establishment

The policy passed in 2019 and was slated for implementation in the 2020 school year, prompting GA DoE to expand the roles of existing staff rather than build a new state-level implementation team from scratch. Initially, two DoE employees—one from the finance department and one contractor—oversaw the implementation efforts, reflecting their roles in both the Synthesis and Translation System (e.g., developing materials and guidance for school staff) and the Support System (e.g., providing technical assistance to frontline implementers). Neither had clearly defined responsibilities or full-time capacity dedicated to the policy.

The finance team member was tasked with developing an allocation formula that reflected legislative intent, namely to improve menstrual material access for low-income students.

…They're taking what's listed legislatively and then they're developing a formula based on what seems to make sense. (State-level implementer)

This staff member continued to lead allocations across subsequent years, creating an internal formula-based process that changed annually based on legislative appropriations and school-level student characteristics. Allocation reports revealed that in the initial program year (fiscal year 2019–2020), all schools serving students in grade 6 or above were eligible to receive funds, with allocations based on half the number of directly certified students—used as a proxy for the number of students who menstruate. Smaller schools received standard amounts ($51 for <50 students, $102 for 50–99 students), while larger schools received scaled allocations based on a calculated factor. This formula remained the same over time, but eligible schools changed three times: in 2020 schools with grades 6–12 with 40% or more students eligible for free or reduced lunch were eligible, in 2021 all schools with grades 6–12 were eligible, and in 2022 and 2023 all schools with grades 5–12 were eligible. The rationale for the first two eligibility changes was not explicitly documented in interviews and was identified through analysis of allocation records; only the final expansion was directly attributed by key informants to GA STOMP advocacy efforts. While eligibility criteria changed and total legislative appropriations increased over time, the average funding per eligible student remained relatively stable between 2021 and 2024 at approximately $2.70 per student (Table 3).

**Table 3:** GA MHH policy allocations over time.

Fiscal year	Total appropriations	Eligible students	Funds per student^a^
2019–2020	$960,000	115,805	$8.29
2020–2021	$300,000^b^	238,530	$1.26
2021–2022	$1,250,000	485,932	$2.57
2022–2023	$1,450,000	526,731	$2.75
2023–2024	$1,450,000	521,495	$2.79

a Funds per student among eligible schools and grades.

b Limited funds due to pandemic cuts, but had midyear governor additions of $720,000 not represented in this table due to lack of allocations data availability.

Beyond the allocations process, the finance staff member held loosely defined responsibilities for outlining allowable purchases, disseminating information to district- and school-level nurses, and offering technical assistance. However, their limited capacity and lack of direct experience with school health needs presented challenges. A contracted employee, affiliated with Children's Healthcare of Atlanta (an institution well-regarded for its school nurse training programs) supported policy roll-out through outreach and webinars, fulfilling functions aligned with the ISF Synthesis and Translation System.

To supplement initial limited state-level capacity, GA STOMP and GASN provided informal but critical implementation support. Their efforts helped bridge gaps between state-level guidance and the day-to-day realities faced by school-level implementers, consistent with the ISF Support System by reinforcing the connection between guidance and practice.

A significant shift occurred in 2022, when DoE hired a full-time staff member with school nursing expertise to manage the policy's implementation through the Office of Whole Child Support. This individual coordinated closely with the finance team and worked to enhance communication, technical assistance, and policy adaptation effort, representing a formalized ISF Support System role.

[Allocations are] all done over in the budget side of the agency. Then, when it's sent over to [the Office of Whole Child Support], they provide the notification of the funds going out so that districts are aware that it's coming, when it's gonna be there. They're supposed to communicate about what's allowed, what's not allowed … They're providing a lot of technical question and answers. (State-level implementer)

This staff member became a central connector across state, district, and school levels—engaging with GA STOMP, GASN, Children's Healthcare of Atlanta, local administrators, and frontline nurses. Their hiring marked a turning point from an initially *ad hoc* implementation approach to a more formalized and sustainable support structure within DoE, ensuring the policy remained responsive to practice-based needs.

I think a major milestone would have to be that there is a full-time person at DoE who is over this, and she cares very much about this program and has shepherded it through and asked good questions and takes every opportunity to explain it to nurses at conferences. (MHH advocate 1)

The establishment of a dedicated, full-time employee institutionalized implementation leadership and reinforced the ISF Support System by ensuring consistent communication, technical assistance, and coordination across sectors.

#### 3.2.2 District- and school-level team and plan establishment

At the district and school levels, administrators were essential members of implementation teams, serving as liaisons between the DoE and their own staff and students. Superintendents and other key administrators (e.g., finance personnel) were informed of the policy and told to prepare for the new funding stream, reflecting their ISF Delivery System roles. The exact administrative team involved in implementation differed based on the structure and size of the district. For example,

…in larger districts that finance person might be like a school nurse finance person for the whole district. But in smaller districts, it's just a finance person that then divides it up … (MHH advocate 1)

In addition to being charged with handling the state-allocated funds, district finance staff were also tasked with submitting fiscal year completion reports to DoE to report if the total allocated amount was spent and return any unspent funds each year.

Recognizing that school nurses were well-positioned to serve as frontline implementers, DoE identified them as the primary actors in on-the-ground policy delivery. As a result, lead nurses and school-based nurses were integrated into implementation teams (i.e., the ISF Delivery System). However, the district- and school-level teams varied across districts based on their size and existing structures. For example, some districts had many nurses, including a lead nurse who supervised multiple school-based nurses while others had a single nurse for the entire district. Implementation processes—including how funds were divided amongst schools and how ordering was done—similarly varied and could be tailored to the individual district's structure and needs.

…Districts receive the funds and are able to work with the schools that have received designated funding in their area to either support them through district level purchasing or just simply pass along those funds and then the schools themselves purchase the products … that is probably different based on different schools and school districts. (MHH advocate 2)

Districts also had the option to either use the exact school-based budget that DoE created (e.g., a school allocated $100 by DoE could only access that exact amount for the year) or keep their funds as one lump sum for schools to use, as needed.

### 3.3 Ongoing implementation support strategies

The full-time staff member who managed the policy's implementation through the Office of Whole Child Support beginning in 2022 established ongoing implementation support strategies, largely consisting of targeted communication, technical assistance, and collaboration with GA STOMP on capacity building and gathering feedback. These activities connected guidance and resources from the state to frontline implementers and facilitated continuous learning across the system (ISF Support System).

#### 3.3.1 Targeted communication

Prior to the employee's onboarding, there were no resources for nurses or administrators to learn about the policy parameters.

The only communication that had been sent out by GA DoE was the webinar and that was during Covid. If you missed the webinar, there wasn't anything in writing that a district could put their hands on to know what was allowed and what was not allowed … so I feel like that was a huge milestone because creating the flyer that gave them some parameters of what's allowed and what's not allowed really helped to answer a lot of questions that everybody had. (State-level implementer)

In addition to the flyer, the DoE employee established an annual communication strategy to first inform superintendents and then lead nurses that are part of the ISF Delivery System about the allocated funding, timeline for receiving funds, and allowable items. These communications provided concrete guidance on allowable and non-allowable expenditures. For example, during the 2022–2023 school year, allowable purchases included menstrual pads, tampons, pantiliners, disposable wipes, and underwear, while items such as soap, shampoo, deodorant, pain-relief medication, and dispensers were explicitly prohibited. In the 2023–2024 school year, dispensers were added to the list of allowable items. Superintendents and lead nurses served as liaisons between the DoE and their own staff and students. DoE also followed up with districts every spring to notify them of unspent funds and the deadline for spending those funds for the fiscal year. District finance employees were also contacted about funds, as well as about the completion report requirements each year.

#### 3.3.2 Technical assistance

Technical assistance was provided year-round by DoE and GA STOMP. The full-time DoE staff served as the primary contact for questions about funding, purchasing, and requirements—fulfilling an ISF Support System role. DoE also collaborated with GA STOMP to provide technical assistance and build capacity among nurses. GA STOMP attended events to inform nurses of the funding and allowable purchases. The organization also announced the allocations and purchasing requirements each year on their website to help spread awareness. Lastly, GA STOMP co-hosted a training with Children's Healthcare of Atlanta, which educated nurses on the policy and on the mental health impacts of inadequate MHH and period poverty. These efforts represent Synthesis and Translation System functions, ensuring that evidence, guidance, and best practices were communicated to the Delivery System.

#### 3.3.3 Supportive feedback mechanisms

While there was no formal process evaluation built into the policy or ongoing implementation efforts, informal and strategic feedback mechanisms were developed over time to support adaptation and continuous improvement. GA STOMP played a central Support System role, gathering and elevating feedback from frontline implementers to DoE and the state legislature. Feedback was collected at professional events and through informal surveys with school nurses, particularly regarding funding sufficiency and the need to expand allocation eligibility from grades 6–12 to grades 5–12. These feedback loops helped identify gaps and supported policy refinements.

Initially, the allocation was for grades 6–12, and in 2021, we got the funding expanded to cover 5th graders as well … That was a big deal because it seems like folks who are in leadership were really hearing what we were saying: “girls are getting their periods younger and younger. We need this coverage in elementary schools. Here's feedback from school nurses.” (MHH advocate 2)

GA STOMP synthesized and relayed such feedback to DoE during regularly established meetings because of their collaborative relationship. This information was also communicated to the state legislature during the legislative session each year through meetings with elected officials and an annual Capitol Day.

The full-time DoE staff member also regularly attended GASN events and was actively involved in the school nursing community, using these connections to learn directly from nurses about implementation challenges and resource needs. In 2024, DoE and GA STOMP jointly engaged in additional outreach to other school personnel, including social workers and counselors, to assess awareness of the policy and funding. These efforts revealed gaps in internal district and school communication, prompting further adjustments in communication strategies and training.

We were hearing from external partners that the schools were needing more products, even though we're providing money for them … And then, I was hearing [from nurses] and still do, “We have too much. We have it stockpiled.” But what we found is that most of the time it was not nurses [reaching out to external partners]. It was counselors or social workers. It's as if they didn't even know there was a supply within their own building or within their own district. That was eye opening. Nurses, counselors, and social workers should work closely together. (State-level implementer)

DoE and GA STOMP also collaborated with researchers on a formative project to assess the acceptability and feasibility of installing menstrual material dispensers in schools. This effort reflected a shared commitment to exploring multiple avenues for feedback and continuous improvement (ISF Synthesis and Translation System). While informal, the project helped surface staff perspectives on access and real-world implementation barriers.

I think had [the recommendation to add dispensers to the allowable list] not been so grounded in good research, I don't think [DoE] would have been willing to allow schools to spend however much money on dispensers. It really reflects our understanding of best practices and getting period products and making them available to people who need them in the moment. (MHH advocate 2)

Though not part of a formal evaluation framework, these efforts allowed implementation actors to iteratively refine their strategies.

### 3.4 Improving future applications

Through implementing the MHH policy over five years, stakeholders across state, district, and school levels identified both strengths and areas for improvement to guide future applications. Much of this learning occurred iteratively, through direct practice and feedback loops coordinated by informal implementation partners like GA STOMP (ISF Support System role), who regularly collected and relayed stakeholder input to the DoE and GA legislature.

#### 3.4.1 Learning from experience

Stakeholders described how implementation matured over time through trial and error, and how some schools—especially those already providing menstrual materials—were able to scale or enhance existing practices:

Before the grant, our school supplied these products. I think that we've just been able to kind of broaden that scope … making sure that [students] don't feel like “hey, here's a pad,” but instead feel like “here's supplies that will get you through the day or the next couple of days or the week, if needed.” (Lead and high school nurse)

A key strength of the policy was its flexibility, allowing districts and schools to adapt strategies to their unique contexts. Nurses in particular developed creative solutions to reduce stigma and increase discreet access to menstrual materials (ISF Delivery System role).

Now that we have the products, [students] have access to them at school, but … some school districts require clear bookbags. You know what we did? We got little pouches that say, “be positive,” or “you rock” … to camouflage their products in the bag. So having the ability to be creative to support the whole period movement and taking some of the stigma out makes it just an incredible program that helps to support school nurses. (District-level director of health services 1)

Common adaptations included procurement and distribution methods for ensuring timely and sufficient menstrual material access, storage and communication strategies to reduce stigma and increase awareness, and material selection based on student need and community norms.

#### 3.4.2 Opportunities for improvement

Despite these successes, several areas emerged for improvement: addressing cultural attitudes and expanding menstrual material options, expanding education and confronting MHH stigma, strengthening communication across school staff, and re-evaluating eligibility criteria to include younger students.

Cultural and gendered attitudes about menstruation influenced what menstrual materials were made available to students. While some schools provided tampons alongside pads, others deliberately excluded them due to discomfort among staff and concerns about parental or community backlash:

We have some parents that feel that we're teaching [the kids] to insert and all this other stuff, and so we just keep it safe here … We have male nurses as well and we don't want that, even though it's education to say how to properly use tampons, to be taken out of context. (District-level director of health services 2)

These concerns reflected broader societal discomfort with MHH. Such misinformation and limited access to current scientific information also posed challenges with policymakers, some of whom relied on outdated narratives or personal beliefs when debating allowable menstrual materials and educational content. Specifically, one MHH advocate highlighted how outdated safety narratives, such as fears about toxic shock syndrome, a rare but potentially serious bacterial condition historically associated with prolonged tampon use, continued to surface in legislative debates, further shaping what materials are perceived as appropriate.

We have people in the legislature who persistently want to talk about toxic shock syndrome and the dangers of tampons … I'm just like “that's not an issue anymore. We took those off the market.” But the amount of legislative effort that has been expended to talk about a problem that happens 5 times a year in the whole US … (MHH advocate 2)

These concerns also underscored the limited availability of accessible MHH education and the extent to which education was often informally dependent on school nurses. MHH education in GA schools was limited to a one-day fifth-grade class on “feminine hygiene,” which students could opt out of with caregiver permission or if a caregiver requested it. Otherwise, nurses educated students one-on-one if they came to their office for MHH-related issues.

Lead and school nurses emphasized that evidence-based educational materials and posters could support student learning, normalization, and consistent messaging across schools. They noted that age-appropriate materials could help students feel prepared, reduce stigma, and lessen reliance on individual nurses for guidance.

A lot of times girls, especially 6th and 7th grade girls, they are almost like embarrassed or ashamed. When you're embarrassed or ashamed of something, you're not going to know how to track [your period], you're not going to pay attention to when your period may be coming on. So I think just educating them in a way that normalizes this experience [would help]. (Middle school nurse)

Communication gaps also emerged as a barrier. Inconsistent communication between school administrators, nurses, counselors, social workers, and teachers—as well as DoE's primary focus on nurses as front-line implementers—limited awareness of available resources. One district-level homeless liaison who manages school social workers and one school social worker did not know about GA's MHH policy until being interviewed for the study. As a result, the social workers in those districts used American Rescue Plan funding or turned to community-based organizations to acquire menstrual materials.

Our [non-profit organization] partner is available to provide support. Sometimes the girls request items that aren't necessarily donated so she has a small fund where she can purchase those overnight pads or the underwear that already have the pad in it. She can purchase those things for our girls with heavier flows. (High school social worker)

This also underscores the diverse experiences and needs of students, especially those that experience heavy bleeding and need access to products that are not always available based on current school approaches.

Some school nurses also reported receiving little or no direct communication about the policy, purchasing options, or available funds because state-level guidance was routed through superintendents and lead nurses. A frequently cited recommendation was the creation of a dedicated website or landing page with up-to-date guidance on allowable purchases and funding details, especially one that could be reference throughout the school year. Document reviews revealed that the only online resource was from fiscal year 2022–2023, which some were still using as a resource although the information was outdated.

There was also mixed understanding across and within districts about how funds could be used; for example, whether each school had to use its specific allocation vs. pooling funds across the district for more flexible use. Generally, districts that managed funds centrally and allowed schools to “take what they need,” reported having sufficient funding compared to those that strictly followed the per-school allocation model. In contrast, when schools were bound by their specific allocation and lacked purchasing input, menstrual material access was often more limited.

Lastly, some school staff expressed concern that the grade eligibility criteria exclude younger students who are already menstruating. Although the policy expanded to include fifth grade, some emphasized that menstruation begins earlier for some students, particularly in low-income and racially diverse communities. Because funds were allocated based strictly on DoE enrollment data for eligible grades in recent years, elementary schools often received little to no support.

I know that the elementary schools don't have as much funding because they go by a formula for age, but we have third graders that have cycles. We have 8 years olds. We have second graders that have cycles. So I think the funding on that level needs to be re-evaluated. (District-level director of health services 1)

This illustrates that current eligibility criteria and allocations may not fully capture the range of students who menstruate, underscoring the need for policy and funding mechanisms to be responsive to the onset of menstruation across age groups to ensure all students have access to needed materials.

## 4 Discussion

Our IAU evaluation of GA's MHH appropriations policy revealed the importance of balancing flexibility and accountability to enable statewide implementation across diverse school districts, a particularly salient issue in a state that prioritizes local control. Most US policies to increase menstrual material accessibility in schools are state mandates (21 of 32 states with policies), requiring schools to provide materials. Yet, funding for implementation is only provided in 12 of those states (26). GA's policy illustrates how providing funds without a mandate can facilitate buy-in among administrators, foster a sense of state support for schools, and enable local adaptation (QIF Phase 1: Initial considerations). Findings from this study offer lessons learned and gaps, both for improving implementation across GA and for other states seeking to adjust, develop, or implement a similar policy. There is very little evidence on how state-level MHH policies are designed and what trade-offs they create between flexibility, accountability, and equity. By teasing out the advantages (buy-in, flexibility, dedicated funding stream) and disadvantages (variability, lack of accountability, inequities), this study addresses a novel gap in the literature.

Advocacy played a central role in the passage, implementation, and sustainment of GA's appropriations policy. Prior research demonstrates that advocacy is a critical tool for passing policies, which has led to recent success in the US due to decades of menstrual activism (11, 28, 60, 61). Uniquely, our findings highlight advocacy's role not only in passing GA's policy, but also in sustaining and shaping its implementation (QIF Phase 3: Ongoing implementation support strategies). Two key partners—the state coalition GA STOMP and the professional association GASN—sustained and even expanded appropriations through direct engagement with policymakers, emphasizing the policy's positive impact and the need to extend funding from grades 6–12 to grades 5–12 to align with the decreasing average age at menarche (62). GA STOMP's involvement in implementation efforts also filled critical capacity gaps by providing supplemental support such as webinars, continuing education opportunities, and educational materials when there was limited dedicated state staffing (QIF Phase 2: Creating a structure for implementation; ISF Support System). They additionally established feedback mechanisms that identified challenges and informed policy refinement over time (QIF Phase 4: Improving future applications; ISF Synthesis and Translation System). These findings underscore how advocacy groups can act as implementation champions and intermediaries, complementing formal state structures, particularly in the early stages of policy implementation when administrative infrastructure is still developing.

This study is, to our knowledge, the first rigorous evaluation of the design and implementation of a statewide MHH policy. Our results extend existing literature focused on cities [i.e., New York City (27) and Chicago (29)] where state mandates require menstrual materials in schools, by revealing the unique advantages and trade-offs of appropriations legislation. In GA, the appropriations policy created a dedicated funding stream for schools to purchase menstrual materials (QIF Phase 1: Initial considerations), a critical step many states have not taken. This positioned the state as a supportive rather than punitive partner, facilitating buy-in from administrators (QIF Phase 3: Ongoing implementation support strategies). It also allowed schools to tailor implementation to their local contexts, which has been shown to be essential for school health initiatives given the well-documented limitations of “one-size-fits-all” policies (63–68).

At the same time, limited programmatic detail and guidance led to under-specification, variability in implementation, and inconsistent understanding across settings. Because the policy itself existed as a budget line item, there was uncertainty around the intended purpose, allowable uses of funds, and implementation procedures. This under-specification resulted in variability and confusion at times. Distribution methods and the types of menstrual materials made available differed widely, potentially leading to or reinforcing inequities in access. For example, some schools provided a variety of materials and distributed them in bathrooms, while others only provided menstrual pads and required students to go to the school nurse's office to obtain them. While flexibility supported contextual tailoring, it also risked reproducing inequities, particularly in districts with fewer administrative or communication resources. Communication breakdowns across nurses, administrators, and other staff constrained policy reach—even when funding was available—illustrating how siloed communication channels can limit impact (QIF Phase 3: Ongoing implementation support strategies; ISF Delivery and Support Systems). This tension between flexibility and equity underscores a central implementation challenge for state-led policies operating in decentralized education systems (QIF Phase 4: Improving future applications). It also underscores the advantage of state mandates, which allow for detailed guidelines for uniform implementation, though the actual effectiveness of such guidelines is still not well understood and needs to be examined.

Additionally, sole reliance on a nurse-based implementation model may have limited the reach of GA's MHH policy. In GA, and across the US, it is common for nurses to cover multiple schools and manage competing responsibilities (69), which may constrain consistent implementation. This is particularly relevant in GA schools given that the full-time school nurse-to-student ratio and the school nursing support staff-to-student ratio are both substantially higher than the National Association of School Nurses' recommended ratio of 1:750 (50). Requiring students to approach a nurse for menstrual materials may also result in gatekeeping that can contribute to discomfort, delays, or missed class time (7, 20, 21, 27). Use of localized and diverse distribution approaches (e.g., via bathrooms, teachers, school social workers) while ensuring adequate training, educational resources, and shared responsibility across school staff could therefore improve policy reach and minimize potential gatekeeping.

To strengthen future implementation, policymakers and state-level implementers should provide clear, evidence-based guidance while still preserving local flexibility. Doing so will require research to identify effective policy designs and implementation strategies. While the limited number of effectiveness trials of menstrual material provision and educational interventions have demonstrated improved school attendance, MHH knowledge, and wellbeing, more rigorous research is needed to inform best practices for policy design and implementation (ISF Synthesis and Translation System) (2, 4, 5, 70).

Still, MHH policies that focus solely on providing menstrual materials may be insufficient to enhance the capacity of schools to effectively address the needs of adolescents who menstruate. Key informants emphasized the importance of integrating MHH education into school health curricula, a gap that persists nationally. MHH education remains minimal and inadequate across the US with only three states covering menstrual materials (California, Michigan, and New Jersey) and three addressing menstruation management (Michigan, Oregon, Utah) (71). Beyond access and education, key informants also underscored the need to reduce stigma and better equip school staff to support those experiencing cycle-related discomforts and pain. Furthermore, ensuring that school bathrooms are menstrual-friendly, including facilities that provide privacy for changing products and discreet disposals options, is necessary (60, 72, 73); as are comprehensive strategies to help students manage cycle-related pain, which is a significantly under addressed issue (60). Addressing these gaps through education, training, and stigma reduction efforts could complement material access policies and create a more holistic approach to MHH in schools (QIF Phase 4: Improving future applications; ISF Support and Delivery Systems).

### 4.1 Strengths and limitations

This study used rigorous qualitative methods that strengthen the validity of findings, including analyzing verbatim transcripts, doubling coding, and systematic team debriefings (51, 74, 75). Using KIIs in conjunction with document reviews enabled a more comprehensive assessment of policy implementation than would have been possible with a single data source (76, 77). The KII sample included individuals engaged in diverse implementation activities at multiple levels (i.e., state, district, school) and from different regions across GA, strengthening the breadth and transferability of findings. However, the study did not capture the perspectives of all staff potentially involved in MHH. For instance, we were unable to recruit participants from charter schools or school counselors, and we did not attempt to recruit janitorial or custodial staff, whose experiences may differ from those represented here. Additionally, we did not obtain insights from students themselves, which is a critical missing group that should be prioritized in future research. Nonetheless, participants working at the state and district levels were able to provide insights that contextualized and helped address these gaps. Lastly, reliance on key informants may have biased our findings because they could have provided incomplete or inaccurate information. However, the use of process documents in conjunction with KIIs provided key details that were not reliant on key informant reporting.

## 5 Conclusion

GA's MHH appropriations policy illustrates how providing funds without a mandate can facilitate buy-in among administrators, foster a sense of state support for schools, and enable local adaptation. At the same time, limited programmatic detail and guidance led to under-specification, variability in implementation, and inconsistent understanding across settings. To strengthen future implementation, increased monitoring and evaluation is needed, including gathering feedback from students who are the intended users. Additionally, policymakers and state-level implementers should provide clear, evidence-based guidance while still preserving local flexibility. Doing so will require research to identify effective policy designs and implementation strategies that promote both equity and sustainability. Lessons from GA's experience can inform other states seeking to develop or refine MHH policies that balance flexibility, accountability, and access.

## Data Availability

The datasets presented in this article are not readily available because participants of this study did not agree to their complete data being shared publicly, so full interview transcripts are not available. However, participants did agree to de-identified excerpts or quotes from transcripts being available with publications. Thus, supporting excerpts that constitute the minimal dataset have been provided, as relevant, throughout the manuscript. Requests to access the datasets should be directed to aballard11@gsu.edu.
