# Supplementary material for: “It was a new concept to talk about periods at the state capitol”: a mixed methods implementation-as-usual evaluation of Georgia's menstrual health and hygiene policy
Source: Front Reprod Health. 2026 Jan 12;7:1745263. doi: 10.3389/frph.2025.1745263 (PMC12832718; doi:10.3389/frph.2025.1745263)
Supplement: Supplementary file 1 [file Table1.pdf]

"*It was a new concept to talk about periods at the state Capitol*": A mixed methods implementation-as-usual evaluation of Georgia's menstrual health and hygiene policy

April M Ballard[1*], Emily Wallace[2], Pranitha Kaza[3], Claire Cox[4], Adele Stewart[4], Shannon R Self-Brown[2]

[1] Department of Population Health Sciences, Georgia State University School of Public Health, 140 Decatur Street SE, Atlanta, Georgia, USA
[2] Department of Health Policy and Behavioral Sciences, Georgia State University School of Public Health, 140 Decatur Street SE, Atlanta, Georgia, USA
[3] Georgia Institute of Technology, 625 Techwood Drive NW, Atlanta, Georgia, USA
[4] Georgia Stop Tax on Menstrual Products, P.O. Box 67, Macon, Georgia, USA

I. Good Reporting of a Mixed Methods Study checklist

Item #	Guideline	Location where item is reported
1	Justification to use a mixed methods approach to the research question	Methods: *Key informant interviews*; Methods: *Document reviews*
2	Articulation of the design in terms of purpose, priority, and sequence of methods	Methods: *Study design and conceptual basis*; Methods: *Document reviews*; Methods: *Data analysis*
3	Describe each method in terms of sampling, data collection, and analysis	Methods: *Key informant interviews*; Methods: *Document reviews*; Methods: *Data analysis*
4	Delineate where and how integration occurs and who has participated in it	Methods: *Data analysis*
5	Describe any limitation of one method associated with the presence of another	Discussion: *Strengths and limitations*
6	Describe insights gained from mixing or integrating methods	Methods: *Document reviews*; Discussion: *Strengths and limitations*

II. Consolidated Criteria for Reporting Qualitative research checklist

Item #	Checklist Item	Location where item is reported
**Domain 1: Research team and reflexivity**		
Personal Characteristics		
1	Author who conducted the interview	Methods: *Data collection*
2	Researcher's credentials	S1 File: *Reflexivity statement*
3	Researcher's occupation at the time of the study	S1 File: *Reflexivity statement*
4	Researcher's gender	S1 File: *Reflexivity statement*
5	Researcher's experience and training	Methods: *Data collection*
Relationship with participants		
6	Relationship established prior to study commencement	S1 File: *Reflexivity statement*
7	Participants' knowledge of the interviewer	S1 File: *Reflexivity statement*
8	Interviewer characteristics reported to participants	S1 File: *Reflexivity statement*
**Domain 2: Study design**		
Theoretical framework		
9	Methodological orientation underpinning the study	Methods: *Study design and conceptual basis*; Methods: *Data analysis*
Participant selection		
10	Sampling approach	Methods: *Sample and participant selection*
11	Method of approaching participants	Methods: *Sample and participant selection*
12	Sample size	Methods: *Sample and participant selection*
13	Non-participation information	Methods: *Sample and participant selection*
Setting		
14	Setting/location of data collection	Methods: *Data collection*
15	Presence of non-participants	Not applicable (no others present)
16	Description of sample	Methods: *Sample and participant selection*
Data Collection		
17	Interview guide provided	S1 File: *Semi-structured in-depth interview guide*
18	Repeat interviews	Not applicable (no repeat interviews)
19	Audio-visual recording	Methods: *Data collection*
20	Field notes	Methods: *Data collection*
21	Duration	Methods: *Data collection*
22	Data saturation	Methods: *Sample and participant selection;* Methods: *Data analysis*
23	Transcripts returned to participants	Not applicable (transcripts not returned)
**Domain 3: Analysis and findings**		
Data analysis		
24	Number of data coders	Methods: *Data analysis*
25	Description of the coding tree	S1 File: *Themes and sub-themes with analytic codes*
26	Derivation of themes	Methods: *Data analysis*
27	Software	Methods: *Data analysis*

28	Participant checking	S1 File: *Reflexivity statement*
Reporting		
29	Quotations presented and identified	Results
30	Data and findings consistent	Results
31	Clarity of major themes	Results
32	Clarify of minor themes	Results

III. Reflexivity statement

The training, experience, and characteristics of the research team informed and influenced this qualitative study. All members of the research team identify as women and brought distinct academic and professional backgrounds that shaped study design, data collection, interpretation, and reporting.

Research background:
The interviewer and first author (AMB) is an academic researcher with a PhD in environmental health sciences and extensive experience conducting qualitative and mixed-methods research on community-based interventions, menstrual health and hygiene, and policy. At the time of the study, she was an assistant professor. The senior author (SSB) is a professor with expertise in implementation science, prevention science, and evidence-based intervention delivery. Other team members included one Master of Public Health student (EW), one undergraduate research assistant (PZ), and two co-founders of GA STOMP (CC and AS) – a statewide menstrual health advocacy coalition – who served as community research partners and member checkers.

Experience and training:
The academic members of the team have formal training in qualitative methods, implementation science, and community-engaged research. The student team members received mentored training in qualitative coding and thematic analysis. The GA STOMP co-founders brought deep experiential expertise in advocacy, policy implementation, and statewide coordination related to menstrual health, which enriched interpretation and contextual understanding.

Relationship with participants and prior connections:
The interviewer (AMB) had prior professional relationships with three of the participants before the study, having met through prior research on Georgia's MHH policy. The co-founders of GA STOMP also had existing professional relationships with some key informants through their advocacy and coalition work. These relationships were acknowledged transparently to participants and contributed to establishing trust and rapport during recruitment.

Participants' knowledge of the interviewer:
Before each interview, participants were informed that the interviewer was a university researcher conducting a study to understand the design and implementation of Georgia's menstrual health and hygiene appropriations policy. They were told that the research was independent from state or advocacy entities and that their participation was voluntary and confidential.

Influence of positionality:
The interviewer's background as a public health researcher with prior experience studying policy implementation likely shaped how questions were asked and how emerging findings were interpreted, particularly with respect to system-level processes and implementation challenges. The involvement of GA STOMP co-founders, who were directly engaged in the policy's passage and sustainment, brought valuable insider perspectives but also required reflexive awareness to avoid overemphasizing advocacy narratives. To mitigate potential bias, member checking, analytic memoing, and team debriefing sessions were conducted throughout the analysis to ensure that multiple perspectives informed data interpretation.

Institutional and power considerations:
All team members were affiliated with academic or advocacy institutions in the United States, which may have influenced participants' perceptions of the research. The team recognized potential power differentials between researchers and key informants from state or local education systems. Reflexive discussions were held throughout data collection and analysis to critically examine how the team's positions and assumptions may have influenced interpretation.

IV. Semi-structured in-depth interview guide

Guide for lead and school nurses and directors of health services

Thank you very much for taking the time to speak with me. Your insights are crucial for understanding the features and implementation of the Feminine Hygiene Grant, which supports the provision of menstrual supplies in public schools. The purpose of this interview is to comprehensively understand the program processes, challenges faced, and successful strategies employed to provide products in schools. The themes that emerge from the interview process will inform future planning, but the interviews themselves will be confidential.

Section 1: General context

1. To start, can you give me an overview of your job and how it involves menstrual health generally?
    a. Probe: Can you tell me a little bit about the student population(s) you serve?
    b. Probe: What do you do specifically relate to the Feminine Hygiene Grant in the state of Georgia that allocates money to schools for period products?
2. Can you describe the current landscape of menstrual health and hygiene in your district/school?
    a. Probe: For example, how well are we doing at meeting children's needs, what are we doing well, what are the challenges?

Section 2: Legislation and policy development

3. Once the funding for period products was established in the state of Georgia in 2019, how did the process for receiving and spending funds unfold in your district/school? Please describe the key milestones and challenges faced.
    a. Probe: Were there any specific people or departments that played a significant role when initiating the use of the funds? If so, how were they involved?
    b. Probe: What were the primary goals and objectives of the funds in your district/school, and how were they determined?
    c. Probe: Were there any debates or controversies surrounding the funds or distribution of products? How were these issues addressed?
    d. Probe: Are there any ongoing or current debates or controversies surrounding the funds or distribution of products? If so, what are they and who are the key players in those debates?

Section 3: Implementation and challenges

4. Can you share details about how the Feminine Hygiene Grant works for you? For example, how and when are funds received, what is the process for ordering products, when does it occur, what is the process for deciding what is ordered?
    a. Probe: Who else is involved beyond nurses (e.g., social workers, counselors)?
    b. Probe: What types of menstrual supplies are distributed in your district/school? Do you give out tampons, why or why not?
    c. Probe: How are these supplies distributed and to whom?
    d. Probe: Is there any technical assistance offered related to funding, purchasing, or distributing? If so, who offers technical assistance and how?
    e. Probe: How is the purchasing and distribution of products monitored, and by who? Does your district/school have any other sources of period supplies?
    f. Probe: Are there any current challenges? If so, how could those challenges be overcome?

5. How have these components changed or evolved over time and what informed these changes?
    a. Probe: How has the funding, purchasing, and distribution of products changed over time?
    b. Probe: Were there/are there any notable challenges during the initial purchasing and distribution of products? How were/are these challenges addressed or mitigated?

Section 4: Impact and evaluation

6. How has the Feminine Hygiene Grant funding impacted your school/district? Students? Community?
    a. Probe: How do you feel about the amount of funding received by your district/school?
    b. Probe: How do you feel about the items that are allowed to be purchased with state funds? Are there other things that would be useful to purchase for your district/school related to menstruation?
    c. Probe: Have there been any unexpected outcomes or challenges arising after the purchasing and distribution of products began?
    d. Probe: How is the effectiveness and sufficiency of products being evaluated? Are there any specific metrics or indicators used to measure impact?

Section 5: Best practices and recommendations

7. Based on your experience, what recommendations do you have for others looking to implement similar policies?
    a. Probe: Are there specific resources or support systems that would be beneficial for other states or organizations working on similar initiatives?
8. What else is needed to improve menstrual health and hygiene in your district/school? In the state of Georgia?
    a. Probe: What do you think is missing from the existing Feminine Hygiene Grant program?
    b. Probe: What are the biggest challenges to achieving those improvements?

Section 6: Closing

9. Is there anyone else in your county that would be useful for me to talk to, whether that be other nurses, social workers, maintenance and operations, etc.?
10. Is there any additional information or insight you would like to share?

Guide for social workers and homeless and foster care program liaisons

Thank you very much for taking the time to speak with me. Your insights are crucial for understanding menstrual health and hygiene for adolescents in public schools. The purpose of this interview is to comprehensively understand any menstrual health and hygiene programming and resources offered in schools, challenges faced, and successful strategies employed to provide products in schools. The themes that emerge from the interview process will inform future planning, but the interviews themselves will be confidential.

Section 1: General context

1. To start, can you give me an overview of your job?
    a. Probe: Can you tell me a little bit about the student population(s) you serve?
2. Can you describe how you interface with menstrual health in your job?
3. Can you describe the current landscape of menstrual health and hygiene in your district/school?
    a. For example, how well are we doing at meeting children's needs, what are we doing well, what are the challenges?

Section 2: Provision of products

4. In your role, can you share details about how programming related to menstrual health and hygiene works? For example, how are these resources funded, what is the process for ordering, who decides what is ordered?
    a. Probe: Who else is involved beyond you (e.g., nurses, counselors)?
    b. Probe: What types of products and resources do you provide? Do you give out tampons, why or why not?
    c. Probe: How are menstrual products and other related resources distributed to students?
    d. Probe: What works well with this process and what are some challenges?
    e. Probe: How is the purchasing and distribution of products monitored, and by who?
    f. Probe: How are those products and resources acquired? What budget do they come from?
    g. Probe: Is funding sufficient to cover all of the needs of the students you serve? Why or why not?

5. Who else do you work with in your school and district when it comes to menstrual health and hygiene?
    a. Probe: Have you ever interfaced with the Feminine Hygiene Grant, which provides money to public schools in the state of Georgia for the purchase of products?
    b. Probe: Would such a program be useful for you and your colleagues? Why or why not?
    c. Probe: Are there other programs or resources that could help you meet the menstrual health needs of your students?

6. Is there any additional information or insight you would like to share?

Guide for state advocates and state-level implementers

Thank you very much for taking the time to speak with me. Your insights are crucial for understanding the features and implementation of the Feminine Hygiene Grant, which supports the provision of menstrual supplies in public schools in the state of Georgia. The purpose of this interview is to comprehensively understand the legislative and program processes, challenges faced, and successful strategies employed to provide products in schools. The themes that emerge from the interview process will inform future planning, but the interviews themselves will be confidential.

Section 1: General context

1. To start, can you give me an overview of your involvement in menstrual health and hygiene in Georgia?
    a. Probe: What work do you and your organization/department do with menstrual health and hygiene?
    b. Probe: What do you do specifically related to legislation or Feminine Hygiene Grant in the state of Georgia that allocates money to schools for period products?
2. Can you describe the current landscape of menstrual health and hygiene in the state of Georgia?
    a. Probe: What has the landscape, particularly related to menstrual policy, been like over time? For example, what improvements or challenges have there been?
    b. Probe: What are factors that influence the landscape and policy in Georgia?

Section 2: Legislation and policy development

3. How did the legislative process for the Feminine Hygiene Grant unfold in Georgia? Please describe the key milestones and challenges faced during the development of the policy.
    a. Probe: Were there any specific stakeholders or organizations that played a significant role in shaping the policy? If so, how were they involved?
    b. Probe: What were the primary goals and objectives of the policy, and how were they determined?
    c. Probe: How was legislative allocation determined to be the policy of choice? Was there any sort of needs, resource, fit, or readiness assessment?
    d. Probe: Were there any debates or controversies surrounding the policy? How were these issues addressed?
    e. Probe: Are there any ongoing or current debates or controversies surrounding the policy? If so, what are they and who are the key players in those debates?

Section 3: Implementation and challenges

4. When the policy was first passed, what were the key components of implementation? For example, how was funding decided upon? Were specific staff hired or jobs expanded to assist with implementation? Was there any capacity building? Were trainings conducted?
    a. Probe: Were these components part of the policy or decided after it was passed?
    b. Probe: How have these components changed or evolved over time and what informed these changes?
    c. Probe: Can you share details about the current implementation process, including the roles and responsibilities of different stakeholders? At the state level? District level? School level?
    d. Probe: What are the differences between traditional public versus charter schools?
    e. Probe: Who offers technical assistance and how?
    f. Probe: How is the policy implementation monitored, and by who? What are the mechanisms for feedback from different stakeholders?

g. Probe: How has the policy implementation and its components changed over time?
h. Probe: Were there/are there any notable challenges during the initial implementation of the policy? How were/are these challenges addressed or mitigated?
i. Probe: Are there any current challenges to the implementation of the policy? If so, how could those challenges be overcome?

<u>Section 4: Impact and evaluation</u>

5. How has the Feminine Hygiene Grant impacted schools? Students? Communities?
    a. Probe: Have there been any unexpected outcomes or challenges arising after the Feminine Hygiene Grant was implemented?
    b. Probe: How is the effectiveness of the Grant being evaluated? Are there any specific metrics or indicators used to measure its impact?

<u>Section 5: Best practices and recommendations</u>

6. Based on your experience, what recommendations do you have for other states looking to implement similar programs?
    a. Probe: Are there specific resources or support systems that would be beneficial for other states or organizations working on similar initiatives?
7. What else is needed to improve menstrual hygiene in Georgia?
    a. Probe: What do you think is missing from the existing Feminine Hygiene Grant policy?
    b. Probe: What are the biggest challenges to achieving those improvements?
8. Who are other relevant groups that are engaged in this work in your state?
    a. Probe: What role(s) do they play?
    b. Probe: Who would you recommend that we talk to?

<u>Section 6: Closing</u>

9. Is there any additional information or insight you would like to share?

V. Document review data extraction table

We extracted and mapped content from each document to the relevant ISF systems and QIF phases. When content was directly relevant, excerpts and verbatim text from the documents were included in the corresponding cells to illustrate alignment and support interpretation.

**Document identification and context**					**ISF system mapping**			**QIF phase mapping**				**Relevance**		
**Document ID**	**Document type**	**Date range**	**Audience and purpose**	**Access or source**	**Synthesis and translation system**	**Support system**	**Delivery system**	**Phase 1**	**Phase 2**	**Phase 3**	**Phase 4**	**Interpret-ation**	**Overlap with KII data**	**Notes**
D01														
D02														
D03														
D04														
…														

VI. Themes and sub-themes with analytic codes

Theme	Definition	Sub-theme(s)	Parent codes (child codes)
Implementation system structure and function (ISF)	Describes the multi-level system through which GA's MHH policy was delivered, supported, and sustained, including organizational relationships, contextual influences, relational dynamics, and feedback mechanisms (following the ISF framework)	• Delivery system<br>• Support system<br>• Synthesis and translation system<br>• Outer context<br>• Inner setting	• System (Delivery system, Support system, Synthesis and translation system)<br>• Individuals (Implementation deliverers, Implementation facilitators, Opinion leaders, High-level implementation authority)<br>• Outer context (Funding resources, Local attitudes, Political)<br>• Inner setting (Relative priority, Connections, Infrastructure)
Implementation phases and processes (QIF)	Captures the temporal and procedural stages of MHH policy implementation, following the QIF framework from initial considerations to ongoing improvement	• Phase 1: Emergence of policy and initial considerations<br>• Phase 2: Creating an implementation structure<br>• Phase 3: Ongoing implementation support strategies<br>• Phase 4: Improving future applications	• Phase 1: Initial considerations (Assessment strategies, Decisions about adaptation, Capacity-building strategies)<br>• Phase 2: Creating an implementation structure (Creating an implementation team, Developing an implementation plan)<br>• Phase 3: Ongoing implementation support strategies (Technical assistance, Process evaluation, Supportive feedback mechanism)<br>• Phase 4: Improving future applications<br>• Evaluation (Impact, Solutions, Challenges)
Core implementation components	Encompasses the tangible and procedural elements of policy enactment and delivery, including menstrual materials, education, and related supports.	• Purchasing and distribution of menstrual materials<br>• Education and communication<br>• Supportive social environment	• Process and components (Needs, Impacts, Supportive social environment, Discomfort, Education, WASH, Menstrual materials, Distribution, Purchasing)<br>• Outer context (Funding resources, Local attitudes, Political)
